# Supplementary material for: Endogenous memory reactivation during sleep in humans is clocked by slow oscillation-spindle complexes
Source: Nat Commun. 2021 May 25;12:3112. doi: 10.1038/s41467-021-23520-2 (PMC8149676; doi:10.1038/s41467-021-23520-2)
Supplement: Supplementary file 1 — Supplementary Information [file 41467_2021_23520_MOESM1_ESM.pdf]

Supplementary Information for:

Endogenous memory reactivation during sleep in humans is clocked by slow oscillation-spindle complexes

Thomas Schreiner[1], Marit Petzka[2], Tobias Staudigl[1] & Bernhard P. Staresina[2*]

1 Department of Psychology, Ludwig-Maximilians-University Munich, GERMANY
2 School of Psychology and Centre for Human Brain Health, University of Birmingham, UK

Correspondence: b.staresina@bham.ac.uk

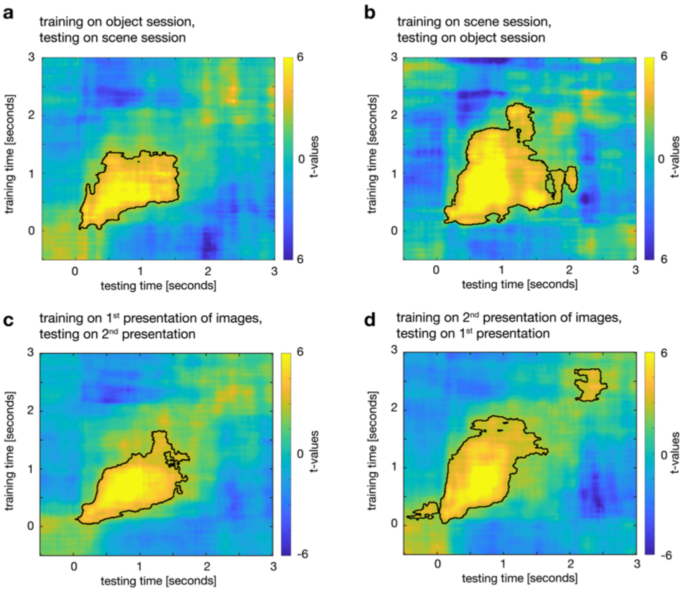

Supplementary Fig.1. Stability of EEG object vs. scene decoding during the localizer tasks. (a + b) To estimate the across-session stability of our decoding approach, we trained a classifier on the localizer data of object sessions and tested it on the localizer data of the scene sessions. **(a)** We found robust above-chance classification when training the classifier on the localizer of the object session and applying the training weights to the corresponding data from the scene session (two-sided dependent-samples t-test; $p_{cluster} = 0.002$, cluster corrected across time). **(b)** The same result pattern emerged when training on the scene session data and applying the training weights to the object session data (two-sided dependent-samples t-test; $p = 0.002$, cluster corrected across time). **(c + d)** During the localizer task, each image was presented twice. To estimate the across-presentation stability of our decoding approach, we trained a classifier on the first image presentation tested it on the second presentation. **(c)** We observed a significant cluster of above-chance classification when training the classifier on the first presentation and applying it to the second presentation (two-sided dependent-samples t-test; $p_{cluster} = 0.0018$, cluster corrected across time). **(d)** A highly comparable result pattern emerged when training on the second presentation and applying it to the first presentation (two-sided dependent-samples t-test; $p_{cluster1} = 0.002$; $p_{cluster2} = 0.019$, cluster corrected across time).

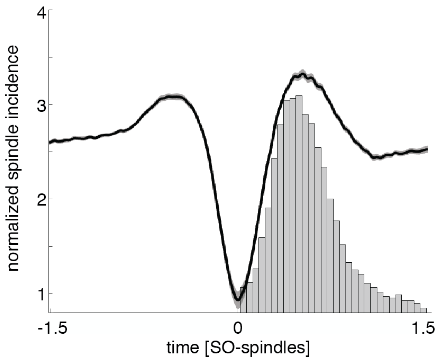

Supplementary Fig.2. Perievent histogram of sleep spindles (amplitude maxima) following SO down-states (time = zero; normalized by number of spindles). Note that due to our definition of SO-spindle complexes (with sleep spindles following SOs), no spindles appear prior to SO down-states (time-point zero). Source data are provided as a Source Data file.

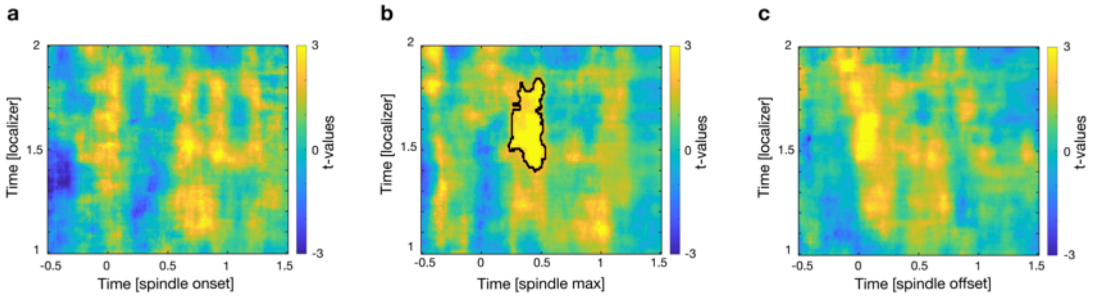

Supplementary Fig. 3. Classification locked to different spindle features. To test for the impact of different features of SO-spindle complexes on the synchronisation of reactivation events, the classification was computed on SO-spindle data locked to the onset, maximum and offset of spindles. Informed by the main analysis, the utilized time-window of both the localizer task and SO-spindle complexes were adjusted accordingly [localizer time: 1000-2000ms; SO-spindle time: -500 to 1500ms relative to respective feature (spindle onset, maximum amplitude and offset)]. Testing accuracy levels against chance at any localizer time x sleep time point for data locked to spindle on-and offsets (a + c) did not lead to any significant above chance classification (two-sided dependent-samples t-test; cluster with the smallest p-value for spindle onset: $p = 0.25$; spindle offset: $p = 0.051$, cluster corrected across time). However, testing the classifier on SO-spindle data locked to the spindle maximum (b) peaks yielded a positive cluster of significant above chance classification (two-sided dependent-samples t-test; $p = 0.019$, SO-spindle time [250 to 500ms], localizer time-window [1400 to 1800ms], cluster corrected across time).

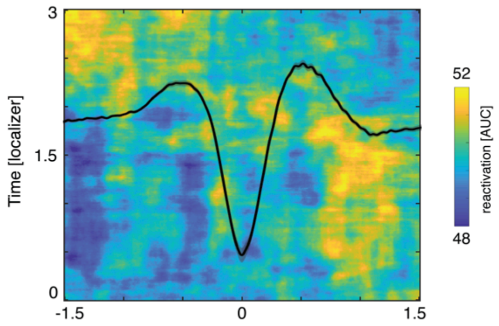

Supplementary Fig. 4. Accuracy map for the classification during SO-spindle complexes. Corresponding accuracy map for the main decoding result reported in Fig. 2b. Color range (blue to yellow) represents decoding performance (Area Under the Curve).

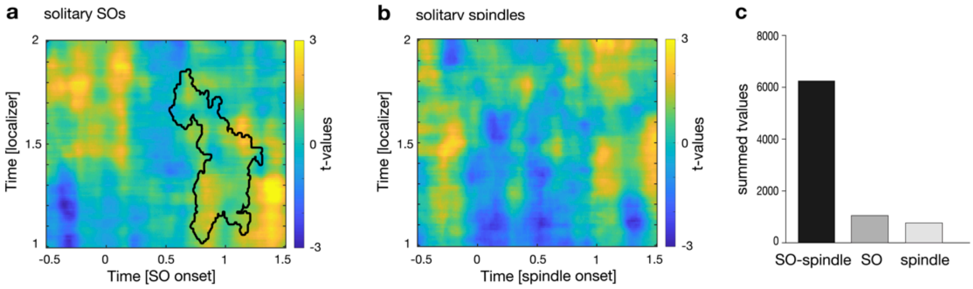

Supplementary Fig. 5. Memory reactivation during solitary SO and spindle events. (a + b). To test whether endogenous memory reactivation indeed requires the joint presence of SOs and spindles, we performed the decoding procedure on solitary SO or spindle events (thus, SOs without spindles and vice versa). Time-windows of both the localizer task and SO-spindle complexes were restricted according to the main results [localizer time: 1000-2000ms; SO-spindle time: -500 to 1500ms relative to respective event (SO down-state, spindle maximum)]. For both types of events, when testing accuracy levels against chance at any localizer time x sleep time point, no significant cluster of above chance classification emerged (two-sided dependent-samples t-test: in both cases cluster with the smallest $p > 0.2$; cluster corrected across time). The black contour lines in (a) illustrate the extent of the significant cluster derived from the main analysis (classification during the presence of SO-spindle complexes). (c) Summed t-values of the significant classification cluster (as derived from the main analysis, corresponding to the black contour lines in Fig 2b and Supplementary Fig 5a) for SO-spindle complexes, solitary SOs and solitary spindles. Source data are provided as a Source Data file.

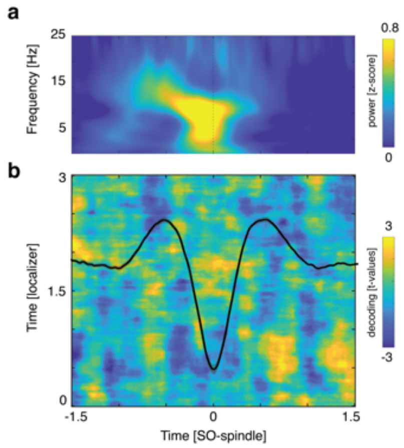

Supplementary Fig.6. SO-slow spindle locked memory reactivation. (a) Time frequency representation of all slow spindle-SO segments (z-scored across time; only positive values are displayed). (b) When testing accuracy levels against chance at any localizer time x sleep time point, no significant cluster of above-chance classification emerged (two-sided dependent-samples t-test; cluster with smallest p value: 0.67, cluster corrected across time). The black line illustrates the averaged EEG trace of all slow spindle-SO segments (electrode Fz).

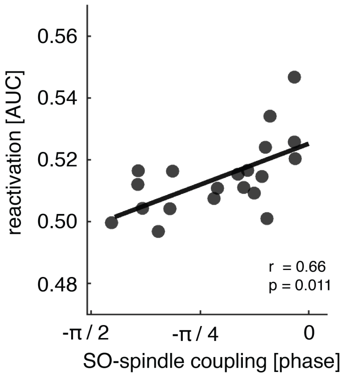

Supplementary Fig.7. Precision of SO-spindle coupling correlates with reactivation strength. Circular-linear correlation analysis between the individual mean SO-spindle coupling phase (circles) and the mean reactivation strength revealed a positive association (r = 0.66; p = 0.011). Source data are provided as a Source Data file.

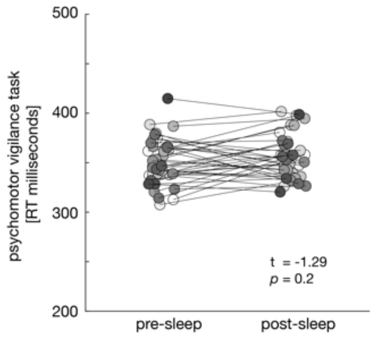

Supplementary Fig.8. PVT results. Before encoding and after the sleep period participants' vigilance state was assessed using a modified version of the psychomotor vigilance task (PVT). During the pre-encoding task the mean reaction time was 350.6 ± 3.6 ms, while the average response time during the post-sleep PVT was 355.2 ± 3.6 ms. Reaction times did not differ between testing times (pre-encoding vs. post-sleep; t = -1.29, p = 0.20). Source data are provided as a Source Data file.

Supplementary Tables

	Objects	Scenes	*t*	*P*
Recognition [Hits] %				
pre-sleep	72.16 ± 4.16	70.91 ± 4.26	0.47	0.64
post-sleep	63.16 ± 4.19	63.58 ± 4.76	-0.79	0.86
post relative to pre	87.41 ± 2.82	87.97 ± 2.88	-0.16	0.85
Recognition [Correct Rejections] %				
pre-sleep	90.00 ± 2.14	85.33 ± 4.48	1.13	0.27
post-sleep	88.00 ± 2.89	83.33 ± 5.22	0.98	0.33
post relative to pre	97.49 ± 1.75	97.33 ± 3.60	0.04	0.96
Recognition [d']				
pre-sleep	2.11 ± 0.14	2.02 ± 0.22	0.47	0.64
post-sleep	1.76 ± 0.19	1.69 ± 0.23	0.37	0.71
Associative Memory %				
pre-sleep	49.16 ± 4.87	46.75 ± 3.08	1.13	0.41
post-sleep	40.08 ± 4.94	36.50 ± 4.46	1.11	0.28
post relative to pre	76.52 ± 5.27	72.61 ± 4.51	0.71	0.48
Associative Memory [out of hits] %				
pre-sleep	64.90 ± 3.99	63.72 ± 5.20	0.33	0.73
post-sleep	59.39 ± 5.71	55.82 ± 5.47	-1.03	0.31
post relative to pre	86.95 ± 5.52	82.55 ± 4.84	0.50	0.55

Table 1. Overview of memory performance. Associative memory % refers to the percentage of correctly recalled images (relative to the total number of stimuli), while associative memory [out of hits] refers to the percentage of recalled image exemplars out of correctly recognized verbs. Statistical differences between conditions (objects vs. scenes) were assessed using dependent samples t-tests (two-sided). Source data are provided as a Source Data file.

Sleep stage [%]	Objects	Scenes	*t*	*P*
N1	2.9 ± 1.6	13.5 ± 2.1	-0.6	0.53
N2	39.5 ± 2.6	48.1 ± 3.1	-1.7	0.09
SWS	22.9 ± 3.3	19.4 ± 2.6	-1.2	0.25
REM	21.9 ± 3.4	16.7 ± 2.8	1.4	0.15
WASO	2.3 ± 0.9	1.4 ± 0.7	0.7	0.49
Total Sleep Time [min]	102.6 ± 3.4	100.6 ± 2.9	0.6	0.53
# spindles	184.8 ± 12.9	188.9 ± 17.9	-0.1	0.85
Spindle density	2.9 ± 0.1	2.82 ± 0.1	0.3	0.74
Spindle duration	0.81 ± 0.01	0.78 ± 0.01	1.3	0.19
Spindle frequency	14.02 ± 0.1	14.06 ± 0.1	-0.2	0.81
# SOs	445.5 ± 31.2	474.1 ± 43.8	-0.5	0.59
SO density	6.9 ± 0.25	7.1 ± 0.32	-0.4	0.68
SO duration	1.38 ± 0.01	1.38 ± 0.01	0.5	0.61
#SO – spindle comp. range	49.4 ± 3.6 [25-88]	50.7 ± 4.9 [12-89]	-0.3	0.79

Table 2. Sleep characteristics. Data are means ± s.e.m. N1, N2: NREM sleep stages N1 & N2, SWS: slow-wave sleep, REM: rapid eye movement sleep, WASO: wake after sleep onset. Statistical differences between conditions (objects vs. scenes) were assessed using dependent samples t-tests (two-sided). Source data are provided as a Source Data file.

P1	0.501
P2	0.497
P3	0.517
P4	0.547
P5	0.510
P6	0.504
P7	0.507
P8	0.534
P9	0.515
P10	0.504
P11	0.508
P12	0.499
P13	0.520
P14	0.511
P15	0.515
P16	0.526
P17	0.512
P18	0.511
P19	0.524
P20	0.516

Table 3. Participant-specific decoding performance averaged across the significant cluster of localizer – SO-spindle classification.

Supplemental Notes

Assessing the impact of trait-like characteristics on the interplay of memory reactivation with the preferred SO-spindle phase and the behavioral expressions of consolidation.

Our results suggest that memory reactivation is linked to the preferred SO-spindle phase as well as to behavioral expressions of consolidation. However, SO-spindle coupling and consolidation might also be governed by other, trait-like participant characteristics. We thus examined - using hierarchical regressions - whether subjective sleep quality (as determined the Pittsburgh Sleep Quality Index (PSQI)) or circadian rhythm (derived from the Morningness-Eveningness Questionnaire (MEQ)) would account for memory performance or the preferred phase of SO-spindle coupling above and beyond reactivation strength during sleep.

In Step 1, reactivation strength explained 29.5% of the variance in memory performance (R^2 = 0.295, $F_{1,18}$= 7.51, P = 0.013). In Steps 2 and 3, neither subjective sleep quality (PSQI global score collapsed across the two sessions, range = 2-5 across participants), nor circadian rhythm (MEQ score collapsed across the two sessions, range = 31-66 across participants) explained significant amounts of additional variance (Step 2: ΔR^2 = 0.001, $\Delta F_{2,16}$= 0.014, P = 0.91; Step 3: ΔR^2 < 0.01, $\Delta F_{3,13}$< 0.01, P = 0.98). Similarly, using the preferred phase of SO-spindle coupling as the dependent variable, in Step 1 reactivation strength explained 37.2% of the variance (R^2 = 0.372, $F_{1,18}$= 10.65, P = 0.004). In Step 2 and 3, neither subjective sleep quality nor circadian rhythm explained significant amounts of additional variance (Step 2: ΔR^2 = 0.07, $\Delta F_{2,16}$=

0.18, P = 0.67; Step 3: ΔR^2 = 0.01, $\Delta F_{3,13}$= 0,30 P = 0.59). Please note that the distribution of the preferred phase values (all clustering between –π / 2 and 0 degrees) enabled us to add these circular data to the linear regression analysis. Nevertheless, to further test for potential associations between subjective sleep quality, circadian rhythm and the preferred phase of SO-spindle coupling, we administered additional circular-linear correlations. Neither sleep quality (rho = 0.04, p = 0.98) nor circadian rhythm (rho = 0.24, p = 0.47) correlated significantly with the preferred phase.

Together, although other trait-like characteristics not captured in the present analysis might have an influence, these results rule out that the link between memory reactivation and consolidation or the phase of SO-spindle coupling is driven by participants' subjective sleep quality or circadian rhythm.
