# Supplementary material for: Endogenous memory reactivation during sleep in humans is clocked by slow oscillation-spindle complexes
Source: Nat Commun. 2021 May 25;12:3112. doi: 10.1038/s41467-021-23520-2 (PMC8149676; doi:10.1038/s41467-021-23520-2)
Supplement: Supplementary file 2 — Reporting Summary [file 41467_2021_23520_MOESM2_ESM.pdf]

nature research

Corresponding author(s): Bernhard Staresina
Last updated by author(s): Mar 24, 2021

Reporting Summary

Nature Research wishes to improve the reproducibility of the work that we publish. This form provides structure for consistency and transparency in reporting. For further information on Nature Research policies, see our Editorial Policies and the Editorial Policy Checklist.

Statistics

For all statistical analyses, confirm that the following items are present in the figure legend, table legend, main text, or Methods section.

n/a	Confirmed	
[ ]	[x]	The exact sample size (*n*) for each experimental group/condition, given as a discrete number and unit of measurement
[ ]	[x]	A statement on whether measurements were taken from distinct samples or whether the same sample was measured repeatedly
[ ]	[x]	The statistical test(s) used AND whether they are one- or two-sided<br>*Only common tests should be described solely by name; describe more complex techniques in the Methods section.*
[ ]	[x]	A description of all covariates tested
[ ]	[x]	A description of any assumptions or corrections, such as tests of normality and adjustment for multiple comparisons
[ ]	[x]	A full description of the statistical parameters including central tendency (e.g. means) or other basic estimates (e.g. regression coefficient) AND variation (e.g. standard deviation) or associated estimates of uncertainty (e.g. confidence intervals)
[ ]	[x]	For null hypothesis testing, the test statistic (e.g. *F*, *t*, *r*) with confidence intervals, effect sizes, degrees of freedom and *P* value noted<br>*Give P values as exact values whenever suitable.*
[x]	[ ]	For Bayesian analysis, information on the choice of priors and Markov chain Monte Carlo settings
[x]	[ ]	For hierarchical and complex designs, identification of the appropriate level for tests and full reporting of outcomes
[ ]	[x]	Estimates of effect sizes (e.g. Cohen's *d*, Pearson's *r*), indicating how they were calculated

Our web collection on statistics for biologists contains articles on many of the points above.

Software and code

Policy information about availability of computer code

Data collection	MATLAB 2018b ,Mathworks, https://uk.mathworks.com/;<br>Psychophysics Toolbox Version 3;<br>Brain Vision Recorder (Brain Products, Munich, GER);
Data analysis	MATLAB 2018b ,Mathworks, https://uk.mathworks.com/;<br>SPSS Statistics 26, IBM, https://www.ibm.com/analytics/us/en/technology/spss/;<br>Fieldtrip Toolbox v.09/01/2020, http://www.fieldtriptoolbox.org/;<br>CircStat Toolbox v.1, https://www.jstatsoft.org/article/view/v031i10; MVPA light, https://github.com/treder/MVPA-Light;<br>Code related to the main analyses of the manuscript is available at https://osf.io/gy25u/

For manuscripts utilizing custom algorithms or software that are central to the research but not yet described in published literature, software must be made available to editors and reviewers. We strongly encourage code deposition in a community repository (e.g. GitHub). See the Nature Research guidelines for submitting code & software for further information.

Data

Policy information about availability of data

All manuscripts must include a data availability statement. This statement should provide the following information, where applicable:

- Accession codes, unique identifiers, or web links for publicly available datasets
- A list of figures that have associated raw data
- A description of any restrictions on data availability

All data supporting the findings of this study are publicly available at the Open Science Framework (https://doi.org/10.17605/OSF.IO/GY25U). Source data are provided with this paper. A reporting summary for this article is available as a Supplementary Information file.

Field-specific reporting

Please select the one below that is the best fit for your research. If you are not sure, read the appropriate sections before making your selection.

[x] Life sciences [ ] Behavioural & social sciences [ ] Ecological, evolutionary & environmental sciences

For a reference copy of the document with all sections, see nature.com/documents/nr-reporting-summary-flat.pdf

Life sciences study design

All studies must disclose on these points even when the disclosure is negative.

Sample size	Sample size was set to 20 participants based upon previous sleep and memory studies (Ngo et al, 2015; Helfrich et al, 2018).
Data exclusions	In the experiment, five participants had to be excluded due to insufficient sleep (less than 30 minutes sleep during one of the sessions; object learning condition: 3 participants; scene learning condition: 2 participants).
Replication	Findings replicated across multiple subjects, and findings also replicated from multiple sessions within some subjects. The experiment was performed once.
Randomization	Statistical controls and cross-validation were used when applicable. Replication mainly comprised determining whether the reported effects were present across participants.
Blinding	The experimenters were not blinded to session allocation, as this was not relevant to the study. There were no differences in outcomes expected, safely excluding any potential bias.

Reporting for specific materials, systems and methods

We require information from authors about some types of materials, experimental systems and methods used in many studies. Here, indicate whether each material, system or method listed is relevant to your study. If you are not sure if a list item applies to your research, read the appropriate section before selecting a response.

Materials & experimental systems

n/a	Involved in the study	
[x]	[ ]	Antibodies
[x]	[ ]	Eukaryotic cell lines
[x]	[ ]	Palaeontology and archaeology
[x]	[ ]	Animals and other organisms
[ ]	[x]	Human research participants
[x]	[ ]	Clinical data
[x]	[ ]	Dual use research of concern

Methods

n/a	Involved in the study	
[x]	[ ]	ChIP-seq
[x]	[ ]	Flow cytometry
[x]	[ ]	MRI-based neuroimaging

Human research participants

Policy information about studies involving human research participants

Population characteristics	All participant demographic information was added to the Methods section. In brief, twenty healthy, right-handed participants (mean age: 20.75 ± 0.35 ; 17 female) took part in the experiment.
Recruitment	Participants were recruited online via University of Birminghams' SONA system (https://www.sona-systems.com).
Ethics oversight	The study was approved by the University of Birmingham Research Ethics Committee.

Note that full information on the approval of the study protocol must also be provided in the manuscript.
